# ANEURYSMAL BONE CYST: A CASE SERIES OF AN AGGRESSIVE BENIGN TUMOR

**DOI:** 10.1590/1413-785220263401e283136

**Published:** 2026-02-13

**Authors:** Caio Falk Giannotti, Nathalia Sundin Palmeira de Oliveira, Jairo Greco Garcia, Marcelo de Toledo Petrilli, Dan Carai Maia Viola, Reynaldo Jesus Garcia

**Affiliations:** 1 Universidade Federal de Sao Paulo, Departamento de Ortopedia e Traumatologia (UNIFEP/EPM), Sao Paulo, SP, Brazil.; 2 Hospital Universitario Pedro Ernesto, Departamento de Ortopedia Oncologica, RJ, Brazil.; 3 Hospital Israelita Albert Einstein, Grupo de Ortopedia Oncologica, Sao Paulo, SP, Brazil.

**Keywords:** Calcitonin, Child, Adolescent, Neoplasms, Benign Neoplasm, Calcitonina, Criança, Adolescente, Neoplasia, Neoplasia Benigna

## Abstract

**Objective:**

The Aneurysmal Bone Cyst (ABC) is a benign yet aggressive bone tumor. This study aimed to evaluate sex, age, tumor location, tumor size, type of treatment (surgical, infiltration, embolization), and recurrence.

**Methods:**

Descriptive and quantitative statistical analyses were applied. The prevalence ratio and 95% confidence interval were calculated for the association between recurrence and sex, age, tumor size, Capanna's classification, and treatment type.

**Results:**

Twenty-three cases of ABCs were included, eleven (47.8%) females and twelve (52.2%) males. The mean age of treated patients was 11.2±1.8 years. Most cysts were located in the lower limbs (56.5%). The mean follow-up time was 42.8±14.01 months. The mean cyst diameter at the beginning of treatment was 5.58± 1.04 cm; of these, 17.4% were up to 3 cm, 43.5% from 3.1 to 6 cm, and 39.1% over 6 cm. Regarding initial treatment, 6 (26%) patients received infiltration, and in total 20 (86.9%) underwent surgery with bone grafting. The overall recurrence rate was 30.4%. No association was identified between recurrence and the variables studied (p ≥ 0.05). The epidemiological data obtained are consistent with pediatric cohorts reported in the literature.

**Conclusion:**

All evaluated methods are suitable for treating aneurysmal bone cysts. ***Level of Evidence IV; Case Series*.**

## RESUMO

**Objetivo:**

O Cisto Ósseo Aneurismático é um tumor benigno, porém agressivo. O objetivo deste estudo foi avaliar sexo, idade, localização e tamanho do tumor, classificação, tratamento realizado (cirúrgico, infiltração, embolização) e recidiva.

**Métodos:**

Foi aplicada análise estatística descritiva e quantitativa para calcular a razão de prevalência e o intervalo de confiança de 95% entre presença de recidiva e sexo, idade, tamanho, classificação e tipo de tratamento.

**Resultados:**

Foram incluídos 23 casos de COA, sendo onze do sexo feminino e doze do sexo masculino. A média de idade foi de 11,2±1,8 anos. A maioria dos cistos localizava-se nos membros inferiores (56,5%). O tempo médio de seguimento foi 42,8±14,01 meses. O diâmetro médio dos cistos no início do tratamento foi 5,58±1,04 cm; destes, 17,4% tinham até 3 cm, 43,5% entre 3,1 e 6 cm, e 39,1% mais de 6 cm. Quanto ao tratamento inicial, 6 (26%) pacientes receberam infiltração e, ao todo, vinte (86,9%) foram submetidos à cirurgia com colocação de enxerto. A taxa global de recidiva foi 30,4%. Não identificamos associação entre recidiva e as variáveis estudadas (p ≥ 0,05). A epidemiologia observada corresponde às coortes pediátricas encontradas na literatura.

**Conclusão:**

Todos os métodos avaliados são adequados para o tratamento do cisto ósseo aneurismático. ***Nível de Evidência IV; Série de Casos*.**

## INTRODUCTION

The aneurysmal bone cyst (ABC) was first described by Jaffe and Lichtenstein, then known as Jaffe-Lichtenstein Disease.^1^ Previously classified as a pseudotumoral lesion, it was reclassified in 2020 in the World Health Organization's tumor classification compendium as a benign tumor lesion and grouped with giant cell tumor and non-ossifying fibroma as osteoclastic lesions rich in giant cells.^2^ This occurred after the finding that the pathogenesis of ABC originates from the translocation of the USP6 gene, evidenced by in situ hybridization *in situ* by fluorescence (FISH).

This tumor most frequently affects children and young adults, mainly in the second decade of life, with a slight predominance in females.^3^ The lesions preferentially occur in the metaphysis of long bones, predominantly in the femur, tibia, and humerus.^4^ Although rare, they can occur in small tubular bones such as metacarpals, metatarsals, and phalanges.^5–7^ In lesions of the spine and pelvis, they represent greater difficulty for surgical treatment.^8–10^

The radiological characteristic is a lytic lesion, typically eccentric and expansive, with a preference for the metaphyseal region of long bones. Lytic lesions may present cortical thinning and widening of the affected segment.^2,3^ The appearance of the lesions shows marked thinning of the cortex over the site, with minimal bone formation; changes are sometimes better visualized on computed tomography or magnetic resonance imaging.^11^

Although ABC is benign, there may be clinical and imaging characteristics that denote aggressiveness.^10^ Surgical treatment options include simple resection in non-displaceable bones or curettage of the lesion, associated with local adjuvant therapy and filling the tumor bed with bone graft or polymethylmethacrylate.^10,11^ The prognosis after treatment is considered good, although about 20% of cases present recurrence.^10^ A treatment program based on the evaluation of the morphological type and aggressiveness of these tumors is recommended and recognized as the Capanna Classification.^12^ The aim of our study was to evaluate a series of cases of children and adolescents with aneurysmal bone cysts and compare the treatment outcomes.

## METHODS

A retrospective case series study on the aneurysmal bone cyst was conducted. Data were compiled from patients’ electronic clinical and imaging records, and a specific database was created for the study with full protection of patient identity. The study was approved by the institutional Ethics Committee and is registered in the Brazil Platform under the number CAE 25729119.0.0000.5505.

The records of 23 patients who underwent surgical and/or clinical treatment for cystic lesions suggestive of ABC with the first consultation in the service between January 1, 2005, and December 31, 2023, were analyzed.

The inclusion criteria for the study were patients with an anatomopathological diagnosis of aneurysmal bone cyst, aged between 1 and 30 years at diagnosis, treated at the institution, and who agreed to participate in the study, with signed informed consent and assent form. The exclusion criterion was noncompliance by the patient or their legal representative with participating in the study at any time. We used Microsoft Excel (Microsoft Office®) for creating the database and tables, and SPSS® V26 (2019) and Minitab 21.2 (2022) for statistical analysis.

All patients were evaluated according to the epidemiological variables: (1) sex; (2) age; (3) tumor location; (4) tumor size (largest diameter); (5) Capanna radiological classification for ABCs;^12^ (6) type of treatment performed (surgical, calcitonin injection, embolization, or a combination of modalities), (7) follow-up time in months, and (8) recurrence of the lesion. Epidemiological analyses of the studied variables were performed, with description of categorical variables (frequency and percentage) and continuous variables (mean and standard deviation). Fisher's exact test was used in analyses where the smallest frequency studied was less than 5. The prevalence ratio was calculated with a 95% confidence interval.

## RESULTS

Table 1 presents complete data on sex, age, location, size, classification, type of treatment (infiltration, embolization, surgery), and recurrence status.

**Table 1:** Descriptive analysis of the cases.

Cases	Sex	Age	Location	Cyst size (largest measurement)	Capanna classification	Type of treatment	Recurrence
1	F	16	Humerus	4.5 cm	I	C	Yes
2	M	14	Tibia	8.6 cm	III	C	No
3	F	12	Tibia	6.0 cm	II	C	Yes
4	F	12	Tibia	4.6 cm	III	C	No
5	F	7	Fibula	5.0 cm	II	C	No
6	M	6	Column	3.2 cm	II	I + E	No
7	M	17	Fibula	10.6 cm	II	C	No
8	M	8	Metatarsus	2.2 cm	I	C	No
9	M	18	Pelve	6.9 cm	II	I	No
10	F	4	Tibia	4.2 cm	III	C	Yes
11	M	6	Tibia	7.0 cm	II	I	Yes
12	F	8	Femur	7.0 cm	III	I + C	No
13	M	15	Metacarpus	4.5 cm	II	C	No
14	M	15	Column	6.2 cm	II	E + C	No
15	M	6	Metacarpus	2.4 cm	II	C	No
16	F	6	Metacarpus	2.4 cm	II	C	Yes
17	F	8	Talus	4.2 cm	II	I + C	Yes
18	M	13	Tibia	8.5 cm	III	C	No
19	F	15	Femur	11.2 cm	II	I + C	No
20	F	17	Radius	2.8 cm	I	C	No
21	M	14	Clavicle	3.4 cm	II	C	No
22	F	9	Femur	7.5 cm	II	C	No
23	M	12	Humerus	5.5 cm	II	C	Yes

F: Female; M: Male; I: Infiltration; C: Surgery; E: Embolization; I + E: Infiltration plus embolization; I + C: infiltration plus surgery; E + C: Embolization plus surgery

**Figure 1:**
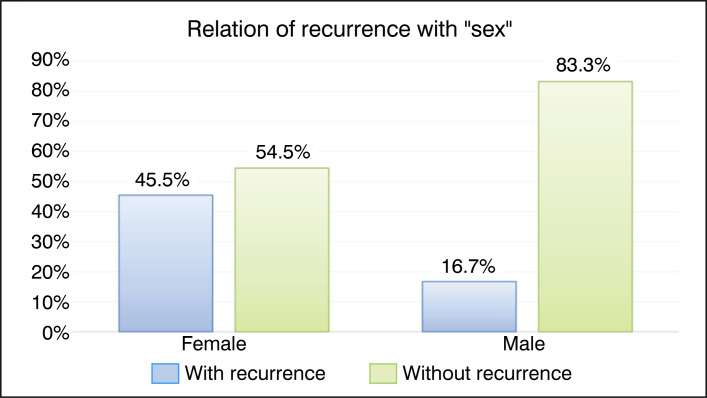
Bar graph of the relationship between recurrence and the qualitative factor "sex".

**Figure 2:**
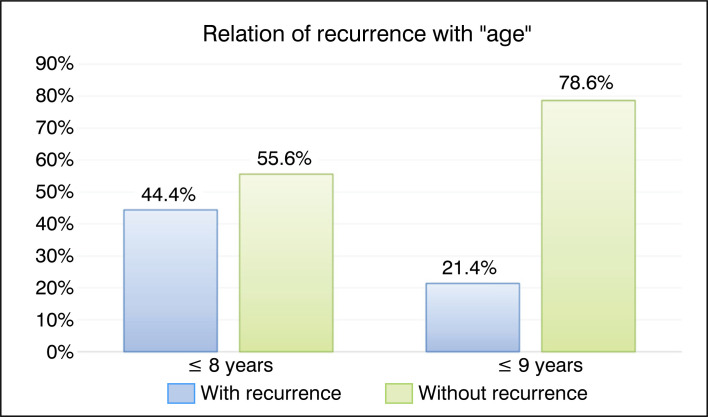
Bar graph of the relationship between recurrence and the qualitative factor "age".

Twenty-three patients were included in the study, with eleven (47.8%) being female and twelve (52.2%) being male. As this is a pediatric hospital, the average age of the treated patients was 11.2±1.8 (4 to 18 years). The graphs in Figures 1 and 2 compare recurrence with the factors "Sex" and "Age", respectively. The results were not statistically significant.

**Figure 3:**
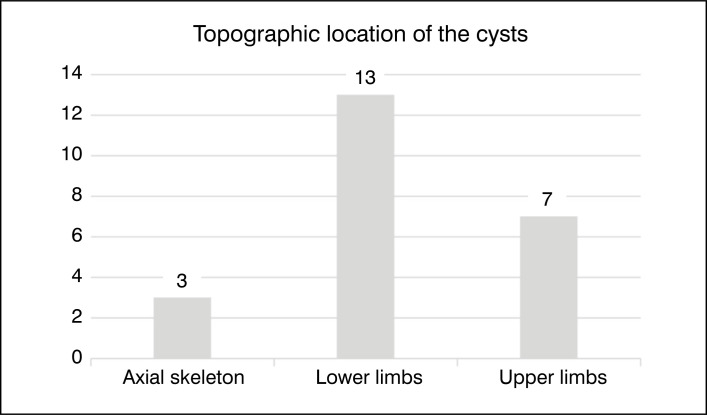
Graph of the distribution of the topographic location of Aneurysmal Bone Cysts: Axial skeleton (pelvis and spine); Lower limbs; Upper limbs.

Regarding the location of the ABC, we observed that the vast majority were located in the lower limbs (56.5%). Figure 3 shows the distribution of the topographic location of the ABCs.

**Figure 4:**
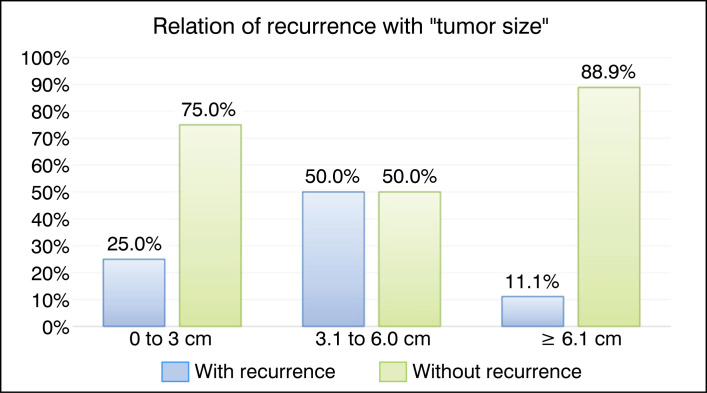
Bar graph of the relationship between recurrence and the qualitative factor "tumor size."

The tumor sizes were evaluated as the average of the largest diameter on the initial treatment radiograph, which was 5.58±1.04 cm, with the smallest 2.2 cm and the largest 11.2 cm. Of these, 17.4% corresponded to cysts up to 3 cm, 43.5% from 3.1 to 6 cm, and 39.1% to cysts above 6 cm. The comparison of these factors with recurrence is illustrated in the graph in Figure 4, but the result was not statistically significant.

**Figure 5:**
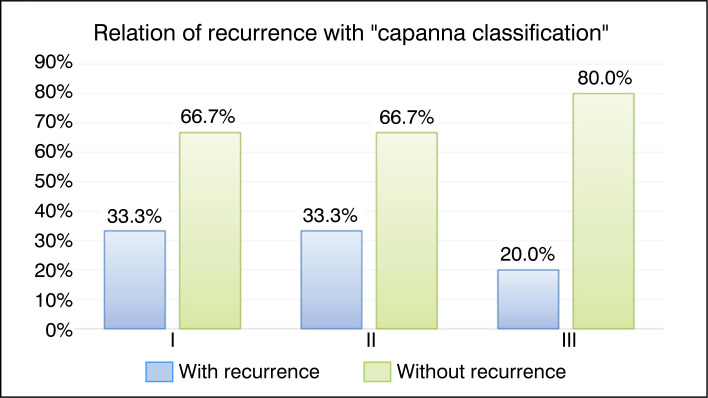
Bar graph of the relationship between recurrence and the qualitative factor "Capanna Classification."

The radiographic classification of Capanna for the ABC was proposed in 1985, based on the radiographic appearance and morphology of these tumors. The classification is divided into five subgroups, as shown in Figure 2. Type I represents lesions centered in the metaphysis, without causing cortical thinning or expansion. Type II involves expansive tumors with cortical thinning that completely involve the affected metaphysis. Type III is the most common type reported by Capanna et al., characterized by an eccentric metaphyseal lesion that typically affects only one cortex. Type IV corresponds to subperiosteal lesions, which grow away from the bone, and type V involves periosteal lesions that expand around the bone and eventually penetrate the cortex below.^12^ Figure 5 presents a comparative graph of recurrence rates for the types of Capanna found in the study patients, with equivalent recurrence rates for types I and II and slightly lower for type III.

Regarding treatment, 6 (26%) patients received infiltration: 5 (83.3%) received calcitonin infiltration, and 1 (16.7%) received dexamethasone and calcitonin infiltration. The dose of each calcitonin infiltration was three ampoules (3 ml) at a concentration of 100 UI/ml. In the case involving the association of calcitonin with dexamethasone, the dose was 2.5 ml at a concentration of 2 mg/ml. Of the patients treated with infiltration, two (33.4%) underwent surgical treatment after recurrence.

**Figure 6:**
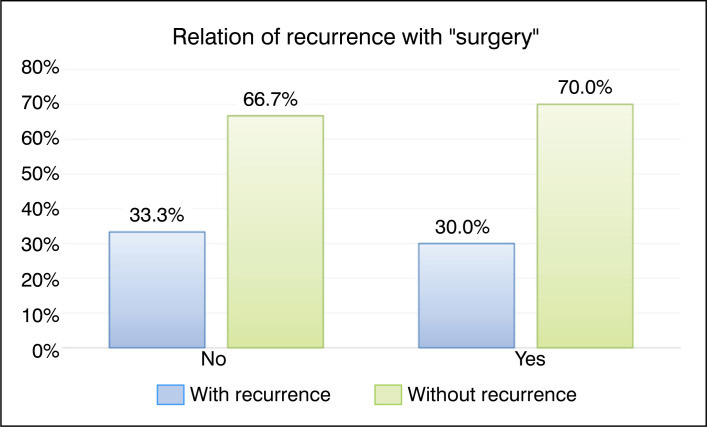
Bar graph of the relationship between recurrence and the qualitative factor "Surgery."

Two patients in total (8.6%) underwent embolization for local control. Both cases were of cysts located in the spinal column, and of them, only one underwent surgery for curettage and grafting at a later time. In total, twenty (86.9%) patients underwent surgery with bone autograft. The surgical treatment of all cases consisted of intralesional resection with extensive curettage, associated with local thermal (electrocautery or Argon scalpel) or chemical (absolute alcohol) adjuvant therapy, followed by grafting in the tumor bed using autograft from the iliac crest. Figure 6 presents a comparative graph of patients who underwent surgery and those who did not, and their respective recurrence rates. Although it shows a slight reduction in the recurrence rate among cases submitted to surgical intervention, the difference is not statistically significant.

The follow-up time was defined as the period from the first consultation in the service to the last consultation included in the study, yielding an average of 42.8±14.1 months (3 to 127 months).

The overall recurrence rate was 30.4% (n=7). We considered the cases that recurred in which there was progression of the size of the radiolucent image in the treated area in two consecutive follow-up X-rays or in which the attending physician reported, in the medical record, the diagnosis of recurrence. In the groups treated with a single method (surgery, infiltration, or embolization), the recurrence rate was 33.4%. In the group that combined the two methods, the recurrence rate among those who started treatment with surgery was 20% (1 in 5 cases). There was no recurrence in the only case initially treated with embolization and then surgery, nor in the only case treated with embolization associated with infiltration, both located in the spine.

Table 2 presents the prevalence and the bivariate analysis of the association between recurrence and the other variables mentioned. We did not identify any association between the investigated variables. Only one patient developed a pathological fracture, 90 months after curettage and grafting. Initially, conservative treatment was attempted with a cast to perform a new curettage and grafting at a later time. The case was re-evaluated, and a new curettage and grafting, followed by internal fixation with plate and screws, was chosen after 17 days of conservative treatment. By the end of this study, the aforementioned patient is three years post-operative with satisfactory consolidation and no signs of recurrence.

**Table 2:** Relation of recurrence with the distribution of qualitative factors: sex, age, tumor size, surgery performed, and Capanna classification (n = 23).

		With Recurrence		Without Recurrence		PR (CI 95%)	P-value
		N	%	N	%		
Sex	Female	5	45.5%	6	54.5%	Ref.	0.124
	Male	2	16.7%	10	83.3%	0.37 (0.10 to 1.36)	
Age	≤ 8 years	4	44.4%	5	55.6%	Ref.	0.187
	≥ 9 years	3	21.4%	11	78.6%	0.48 (0.14 to 1.64)	
Tumor size	0 to 3 cm	1	25.0%	3	75.0%	0.50 (0.10 to 2.45)	0.336
	3.1 to 6.0 cm	5	50.0%	5	50.0%	Ref.	- x -
	≥ 6.1 cm	1	11.1%	8	88.9%	0.22 (0.04 to 1.12)	0.084
Surgery	No	1	33.3%	2	66.7%	Ref.	0.474
	Yes	6	30.0%	14	70.0%	0.90 (0.15 to 5.26)	
Capanna Classification	I	1	33.3%	2	66.7%	Ref.	- x -
	II	5	33.3%	10	66.7%	1.00 (no CI)	0.485
	III	1	20.0%	4	80.0%	0.60 (0.06 to 6.45)	0.536

N: number of individuals; PR: Prevalence Ratio; CI: confidence interval; Ref.: value set as reference for the PR study; "- x -": value for which the statistic is not applicable.

## DISCUSSION

The aneurysmal bone cyst (ABC) is a benign neoplasm rich in giant cells, cystic in appearance, multiloculated, and containing blood.^2^ Although current medical literature presents several works with case series on ABC,^3–7^ there is a lack of studies in Brazil conducted with children and adolescents treated in specialized orthopedic oncology institutions.

In the present study, aneurysmal bone cyst was the predominant lesion in male children and adolescents. This finding differs from the data found in national^13^ and international literature.^14,15^ In fact, most patients diagnosed with ABC are children and adolescents.^13^ ABC can occur in childhood and early adulthood, and there is a slightly increased incidence rate in women (1 to 1.3).^13,15^

In our case series, the cysts were predominantly located in the lower limbs, including the tibia, femur, talus, and metatarsus. Although ABC can occur in any bone, it usually affects the metaphysis of long bones, such as the femur and tibia.^13,15^ It can also affect the vertebrae and pelvis.^13,15^ The diagnosis should be confirmed by biopsy and histopathological evaluation.^15^ ABC is an aggressive benign tumor that often presents with pain, pathological fracture, and may show local recurrence after treatment.^10,13,16^

In this study, most cases of ABC presented radiological classification of Capanna type II, that is, a central expansive lesion affecting the entire bone diameter, promoting cortical thinning. Another study also conducted in Brazil^14^ and international studies also found in their sample a higher prevalence of Capanna type II lesions,^17,18^ although in its original description, Capanna states a higher prevalence of type II.^12^ We did not find in our study, among the included patients, lesions of classification IV or V.

The treatments included infiltration (calcitonin or dexamethasone + calcitonin), embolization for cases involving the spine, and surgery with graft placement. Once ABC is suspected, the patient should be referred to an orthopedic oncologist.^13^ Since it is a bone lesion that may present as active with aggressive and destructive characteristics of the bone, the aneurysmal bone cyst is indicated for intervention. The most frequently performed treatment, according to the literature, is intralesional resection, with curettage of the lesion.^2,11,12^

The use of local adjuvants (phenol, absolute alcohol, electrocautery, argon scalpel) is recommended to optimize tumor cell destruction after curettage, and the affected area should subsequently be filled with bone graft (autologous or homologous) or polymethylmethacrylate (PMMA).^11^ Extensive lesions and/or those involving joints may be treated with marginal or even wide resection, requiring reconstruction of the joint (endoprosthesis) or the affected bone segment.^11^ Endovascular embolization of the arteries supplying the lesion can be performed, aiming to achieve local control in large or surgically difficult-to-access lesions.

In recent years, due to its predominantly osteolytic characteristics and its abundance of multinucleated giant cells resembling osteoclasts, Denosumab has been studied for the treatment of ABC. This medication works by blocking the RANKL receptor, thereby reducing osteoclast activation.^19^ However, studies still do not present a consensus regarding the appropriate dose and duration of treatment. In the present study, the recurrence rate of ABC was 30.4%. By treatment modality, we observed a recurrence rate of 33.4% in patients who received a single treatment (either surgery or infiltration only). In the combined therapy, we observed a 20% recurrence. Moreover, we found no association between recurrence rates and sex, age, or size of the cyst. The recurrence rate after ABC treatment is quite high, in accordance with its aggressive benign nature.

In the literature, recurrence rates after surgical treatment varied from 10 to 59%.^20^ Vergel de Dios et al.^20^ reported that 90% of recurrences occurred in patients under 20 years of age, and the authors associated recurrence with younger age. Still, the statistical analysis did not confirm this association.^20^

## CONCLUSION

The observed epidemiology is consistent with pediatric cohorts reported in the literature. Both surgery and calcitonin infiltration are appropriate treatment options for aneurysmal bone cysts, and embolization can be employed concomitantly or independently of these methods to achieve local control of lesions in surgically difficult-to-access locations. There was no statistically significant relationship between recurrence and the evaluated risk factors, as the value 1.0 was always within the Prevalence Ratio interval.

## Data Availability

The underlying data related to the research are available with the corresponding author.
